# Supplementary material for: Content-rich biological network constructed by mining PubMed abstracts
Source: BMC Bioinformatics. 2004 Oct 8;5:147. doi: 10.1186/1471-2105-5-147 (PMC528731; doi:10.1186/1471-2105-5-147)
Supplement: Additional File 5 — The original Chilibot query results of the term "long-term potentiation (LTP)" and 22 other terms, limiting the latest references analyzed to the years 1990, 1995, 2000, and 2004. [file 1471-2105-5-147-S5.bz2 › chilibotAdditionalFile5/ltp2004/html/PKC_ZIF268.html]

 


 **PKC** and **ZIF268** 
  
Found 6 abstracts in PubMed,  **6 abstracts were retrieved and analyzed**.  


---

 Search Google  |
 PDF files only 
|  EDU domain only 

---

**Interactive relationship** (e.g. stimulation, inhibition, etc)

- Pretreatment of the cells with the specific protein kinase C  **PKC**  inhibitor GF109203X partially inhibits mAChR mediated induction of  **zif268**  mRNA.  Ref: 9048745 J Neurochem, 1997
- These data indicate that both  **PKC**  and the influx of extracellular calcium play a role in the induction of  **zif268**  mRNA following activation of the m1 mAChR.  Ref: 9048745 J Neurochem, 1997

- :-)

  **Parallel relationship** (e.g. studied together, co-existance, homology, etc.)

  - To assess whether physical activity induces alterations in gene expression that may underlie these changes in  **PKC**  and learning performance, we examined the effect of physical activity on expression of  **zif268** , a transcription regulatory factor linked to stimulus induced neuronal plasticity.  Ref: 7889126 Hippocampus, 1994
  - It is possible that diacylglycerols contribute to the induction of  **zif268**  by Pb by sustaining  **PKC**  activation.  Ref: 12587664 Neurochem Res, 2003
  - The mice were then tested on the Morris water maze task for 6 days with subsequent analysis of  **PKC**  activity and  **zif268**  mRNA expression.  Ref: 7889126 Hippocampus, 1994
